# Coronary angiography or not after cardiac arrest without ST segment elevation: A systematic review and meta-analysis

**DOI:** 10.1097/MD.0000000000022197

**Published:** 2020-10-09

**Authors:** Meng-Chang Yang, Wu Meng-Jun, Xu Xiao-Yan, Kevin L. Peng, Yong G. Peng, Ru-Rong Wang

**Affiliations:** a Department of Anesthesiology, West China Hospital, Sichuan University; b Department of Anesthesiology, Sichuan Academy of Medical Sciences and Sichuan Provincial People's Hospital; c Department of Anesthesiology, The Affiliated Hospital, School of Medicine, UESTC Chengdu Women's and Children's Central Hospital, Chengdu, Sichuan, P.R. China; d College Graduate, Rice University, Houston, TX; e Department of Anesthesiology, University of Florida College of Medicine, Gainesville, FL, USA.

**Keywords:** delay or no coronary angiography, immediate coronary angiography, out-of-hospital cardiac arrest, meta-analysis, without ST segment elevation

## Abstract

**Objective:**

This meta-analysis aimed to review the available evidence and evaluate the necessity of immediate coronary angiography (CAG) to obtain positive outcomes for out-of-hospital cardiac arrest (OHCA) patients without ST segment elevation.

**Data sources:**

Web of Science, PubMed, Embase, Chinese National Knowledge Infrastructure, Wanfang, and SinoMed databases.

**Study selection:**

We included observational and case–control studies of outcomes among individuals without ST segment elevation experiencing OHCA who had immediate, delayed, or no CAG.

**Data extraction:**

We extracted study details, as well as patient characteristics and outcomes.

**Data synthesis:**

Six studies (n = 2665) investigating mortality until discharge demonstrated a significant increase in survival benefit with early CAG (odds ratio [OR] = 1.78; 95%CI = 1.51–2.11; *I*^2^ = 81%; *P* < .0001). Seven studies (n = 2909) showed a significant preservation of neurological functions with early CAG at discharge (OR = 1.66; 95%CI = 1.37–2.02; *P* < .00001). Four studies (n = 1357) investigating survival outcomes with middle-term follow-up revealed no significant benefit with early CAG (OR = 1.21; 95%CI = 0.93–1.57; *I*^2^ = 66%; *P* = .15).

**Conclusions:**

Our meta-analysis demonstrates that there may be significant benefits in performing immediate CAG on patients who experience OHCA without ST segment elevation.

## Abstract 2

Supplemental Digital Content is available in the text

## 1 Introduction

Despite advancements in the field of resuscitation and improved management of post-cardiac arrest care, out-of-hospital cardiac arrest (OHCA) remains a leading cause of death in developed nations.^[1]^ The overall prognosis of this patient population continues to be poor.

The most frequent causes of cardiac arrest in post-cardiac arrest patients are ischemic heart disease and coronary artery disease. These two factors are present in up to 70% of all patients who are resuscitated^[1]^ and are key indicators for immediate coronary angiography (CAG) post-cardiac arrest.^[2]^ Current European and American clinical practice guidelines recommend immediate CAG with adjunctive percutaneous coronary intervention (PCI) in patients who present with ST segment elevation myocardial infarction (STEMI) following cardiac arrest.^[3,4]^

For patients experiencing OHCA who present no evidence of STEMI (NSTEMI), the role of immediate CAG is still a matter of debate. The current clinical guidelines from the American College of Cardiology/American Heart Association do suggest emergent angiography in a specific sub-set of NSTEMI patients who are comatose after OHCA and are either hemodynamically or electrically unstable.^[3]^ However, a recent study reported in the *New England Journal of Medicine* by Lemkes^[1]^ suggested that a strategy of immediate CAG revealed no additional beneficial than delayed CAG with respect to overall 90-day survival.

We performed a meta-analysis with current available literature and evaluated the difference in outcomes, including survival and neurological status at discharge, between immediate and delayed CAG for patients who had an OHCA with NSTEMI.

## 2 Materials and methods

### 2.1 Search strategy

Two independent operators (J. Guo J and X.L. Yang) conducted the search using the databases PubMed, Web of Science, Embase, Chinese National Knowledge Infrastructure, Wanfang, and SinoMed. The key words searched included “cardiac arrest,” “OHCA,” “out of hospital cardiac arrest,” “heart arrest,” “coronary angiography,” “coronary angiogram,” “CAG,” “coronary catheterization,” “coronary catheterization,” “PCI,” “percutaneous coronary intervention,” “angioplasty,” “immediate,” “early,” “urgent,” “emergent,” “delayed,” and “late.” This search strategy was further adapted to maximize the acquisition of all pertinent articles for each database searched. The time period of the search was from inception of these databases through July 4, 2019. After exhausting the above-mentioned databases, snowballing from pertinent articles was rigorously performed to ensure that no relevant articles were overlooked. Finally, Grey Literature Databases and Clinical Trials Databases were reviewed as well. All identified articles were compiled using Endnote.

### 2.2 Eligibility criteria

The eligible articles were comprised of randomized controlled trials, cohort studies, and observational studies. For studies reporting outcomes for both STEMI and NSTEMI patients following OHCA, only data pertaining to NSTEMI patients was extracted and applied to the analysis.

### 2.3 Exclusion criteria

Letters to the editor, reviews, case reports, commentaries, duplicates, and conference abstracts were excluded from the analysis following the screening of abstracts by each reviewer. Furthermore, studies that failed to quantitatively describe study outcomes, such as survival, mortality, and neurological status at discharge or follow-up were also excluded. Evaluation of full-text articles for analysis was performed by both reviewers, and any conflict raised over study inclusion was resolved by mutual consensus.

### 2.4 Outcomes

Survival and neurological outcomes were the primary outcomes in our analysis. Survival was determined at hospital discharge and during middle- to long-term follow-up. The time to follow-up was variable between each study and ranged from 6 to 14 months. Neurological outcomes were assessed in terms of cerebral performance category scores. A score of 1 to 2 indicated consciousness with little or no cerebral damage and was considered a good score. These scores were examined at discharge and middle-term follow-up, which was defined as a period of 1 to 3 months. Early CAG was defined differently in every study, ranging from on admission, within 2 h of admission, and between 6 and 12 h after admission. The time to immediate assessment of outcomes was accepted as defined in all studies included. Data from eligible papers were extracted into a predetermined, standardized Excel spreadsheet that recorded study demographic characteristics and the baseline clinical, interventional, and outcome details for the population of interest as Utstein data points.^[5]^ Quality assessment was performed by two reviewers (J. Guo and X.L. Yang) for eight observational studies and two randomized controlled trials.

### 2.5 Statistical analyses

We analyzed the data with Stata 12.0 (StataCorp, College Station, TX). Significance in all analyses was defined as *P* < .05. *I*^2^ was calculated to evaluate the heterogeneity among studies: *I*^2^ < 25% was considered as absence of heterogeneity (homogeneity); 25% ≤ *I*^2^ < 50%, low heterogeneity; 50% ≤ *I*^2^ < 75%, moderate heterogeneity; and *I*^2^ ≥ 75%, substantial heterogeneity.^[6]^ A fixed-effect model was used to meta analyze pooled data classified as homogeneous or of low heterogeneity. A random-effect model was used to meta-analyze data classified as of moderate or substantial heterogeneity.^[7]^ Egger's and/or Begg's tests were used to evaluate publication bias (*P* > .05 indicates no publication bias).^[7,8]^

## 3 Results

### 3.1 Literature search and included studies

**Figure 1:**
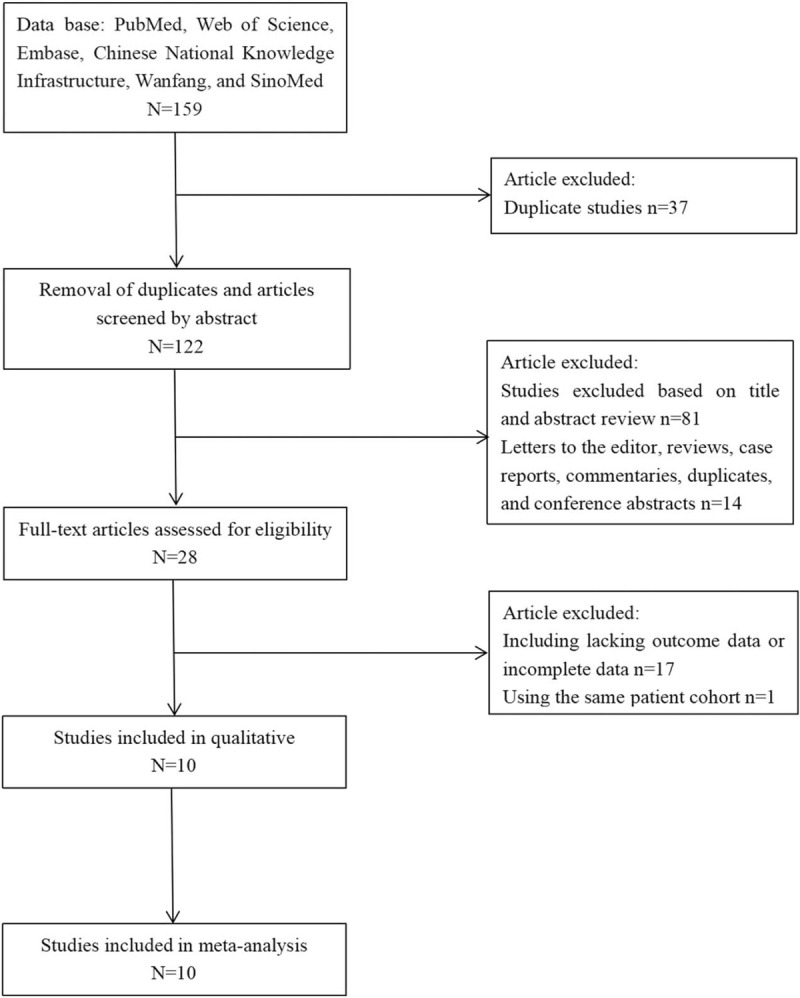
Study selection flow diagram.

The systematic literature search yielded 10 studies that meet the inclusion criteria for this meta-analysis. After searching the six databases and removing duplicates, 224 articles were evaluated in full for eligibility, resulting in the total 10 studies^[1,9–17]^ (early vs delayed or no CAG, n = 1599/2287) in the meta-analysis. Some studies were excluded due to our inability to differentiate between STEMI and NSTEMI subgroups from the heterogeneous population of OHCA patients.^[18–22]^ The detailed literature search can be seen in Figure 1. Baseline demographic and clinical characteristics of all the studies are outlined in Supplemental Table 1 (http://links.lww.com/MD/E927). Of the 10 studies included, eight were observational in nature; seven of these were retrospective^[9,11–17]^ and one was prospective.^[10]^ Two studies were randomized controlled trials.^[1,14]^ Early CAG was defined differently in every study (on admission, within 2 h, or between 6 and 12 h of admission). Patients were followed for 6 to 14 months in most of the studies. The mean follow-up period was 9 months. The mean age of patients at admission was 61 years. PCI was also attempted in most of the patients. However, we observed that a greater number of PCI was performed in patients who underwent early CAG (40%) compared to those who underwent late or no CAG (20%).

### 3.2 Survival to discharge

**Figure 2:**
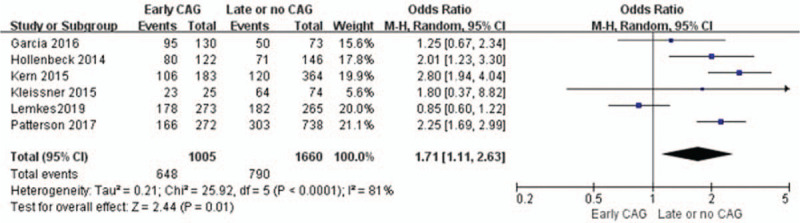
Forest plot of survival to discharge with early coronary angiography and delay or no coronary angiography individuals, based on data from six studies that compared the two types of individuals in parallel. The *x*-axis indicates the 95% confidence interval. ES = effect size.

Six studies (n = 2665) show a significant increase in survival until discharge with early CAG (odds ratio [OR] = 1.78; 95%CI = 1.51–2.11; *I*^2^ = 81%; *P* < .0001; Fig. 2).^[1,9,12,13,15,16]^ However, based on the GRADE (Grading of Recommendations, Assessment, Development and Evaluations) framework, this finding was determined to be low-quality evidence. In these six studies, high heterogeneity was detected (*I*^2^ = 81%; *P* < .0001) in the report of survival to admission among individuals, and a random-effect model was used to further process this data. The funnel plot is visually symmetrical, suggesting no significant observational bias. We obtained a similar conclusion by Egger's test (*P* = .38) and Begg's test (*P* = .259).

### 3.3 Survival until discharge with neurological function

**Figure 3:**
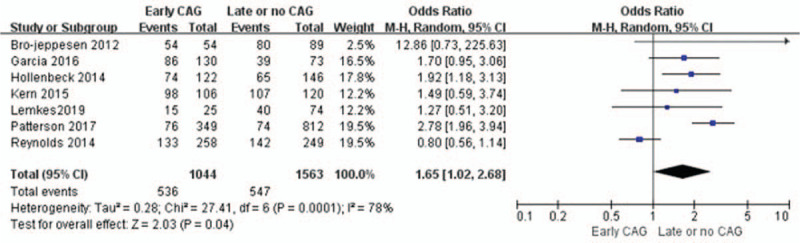
Forest plot of survival to discharge with cerebral performance categories 1 to 2 with early coronary angiography and delay or no coronary angiography individuals, based on data from seven studies that compared the two types of individuals in parallel. The *x*-axis indicates the 95% confidence interval. ES = effect size.

Seven studies (n = 2909) show significant preservation of intact neurological function until discharge with early CAG (OR = 1.66; 95%CI = 1.37–2.02; *P* < .00001; Fig. 3).^[1,9,10,12,13,15,16]^ In these seven studies, which report survival to admission among individuals, high heterogeneity was detected (*I*^2^ = 83%; *P* < .0001), and a random-effect model was used to further process the data. The funnel plot is visually symmetrical, suggesting no significant observational bias. A similar conclusion was achieved by Egger's test (*P* = .881) and Begg's test (*P* = .620).

### 3.4 Survival to middle-term follow-up

**Figure 4:**
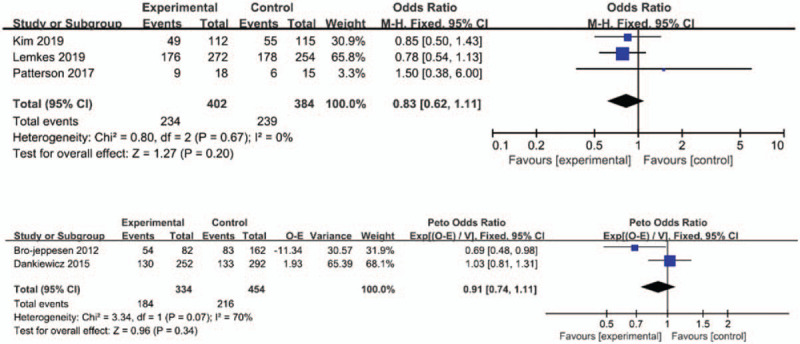
Forest plot of survival to middle term with early coronary angiography and delay or no coronary angiography individuals, based on data from four studies that compared the two types of individuals in parallel. The *x*-axis indicates the 95% confidence interval. ES = effect size.

Five studies (n = 1574) show that there is no significant increased survival after middle-term follow-up with early CAG (OR = 1.21; 95%CI = 0.93–1.57; *I*^2^ = 0%; *P* = .15; Peto OR = 0.91, 95%CI = 0.74–1.11; *I*^2^ = 70%; *P* = .34; Fig. 4).^[1,10,11,14,17]^ We define middle-term follow-up as 30 to 90 days after hospital discharge. Based on the GRADE framework, this finding was considered to as high-quality evidence. In these three studies, which report OR between early CAG and later, absence of heterogeneity was detected (*I*^2^ = 0%; *P* = .67), and a fixed-effect model was used to further analyze the data. The funnel plot is visually symmetrical, suggesting no significant observational bias. A similar conclusion was confirmed by Egger's test (*P* = .497) and Begg's test (*P* = .948).

### 3.5 Survival with middle-term follow-up for neurological function

**Figure 5:**
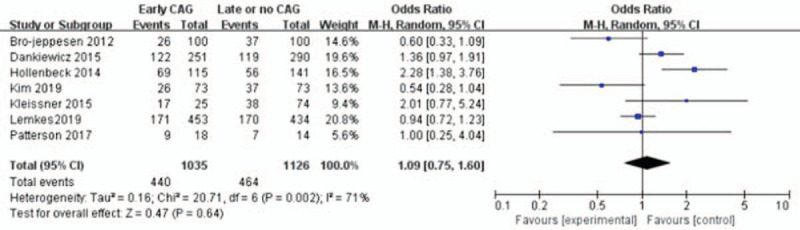
Forest plot of survival to middle term with cerebral performance categories 1 to 2 with early coronary angiography and delay or no coronary angiography individuals from four studies that compared the two types of individuals in parallel. The *x*-axis indicates the 95% confidence interval. ES = effect size.

Four studies (n = 1357) show significant preservation of intact (*P* = .03) neurological function after middle-term follow-up with early CAG (OR = 0.74; 95%CI = 0.59–0.97; Fig. 5).^[1,10,14,17]^ The funnel plot of the overall result is skewed to the right. In these four studies, which report survival with middle-term follow-up for neurological function among individuals, high heterogeneity was not detected (*I*^2^ = 0%; *P* = .54), and a M-H fixed model was used to further analyze the data. A similar conclusion was reached by Egger's test (*P* = .34) and Begg's test (*P* = 971).

## 4 Discussion

This is the most up-to-date systematic review and meta-analysis on patients with NSTEMI undergoing CAG following OHCA. Overall, cerebral performance categories 1 and 2 benefits were conferred by early CAG at hospital discharge and were also seen at middle-term follow-up in the setting of NSTEMI OHCA. Early CAG also seemed to indicate survival benefits at hospital discharge in the setting of NSTEMI OHCA. Therefore, the present study suggests that there is a significant benefit of performing early CAG over delayed or no CAG in patients presenting with NSTEMI following OHCA.

Our findings corroborate the results of previous studies that show a survival benefit at discharge and favorable neurological outcomes both at discharge and middle-term follow-up with early CAG in patients who had OHCA with NSTEMI. Multiple observational studies have demonstrated the survival benefit conferred by an early and successful PCI in the setting of NSTEMI OHCA.^[23–25]^

In 2014, a meta-analysis compared early CAG with conservative management (late/no CAG) in patients with ST elevation as well as no ST elevation.^[26]^ The study reported a survival benefit and good neurologic prognosis with CAG (respectively, OR = 2.77; 95%CI = 2.06–3.72; *P* < .0001 from 15 studies with 3800 patients and OR = 2.2; 95%CI = 1.46–3.32; *P* < .0002 from 9 studies with 2919 patients). Another meta-analysis, conducted in 2012, compared CAG and PCI with conventional treatment (late/no CAG) in patients with and without ST elevation and revealed improvement in survival with early CAG (OR = 2.78; 95%CI = 1.89–4.10; *P* < .001 in 10 studies with 3103 patients).^[24]^ A meta-analysis by Khan et al^[27]^ that included eight studies, with some published as recently as 2017, compared acute CAG with non-acute CAG in patients without ST elevation followed OHCA. The study concluded that early CAG was associated with decreased short-term mortality (OR = 0.46; 95%CI = 0.36–0.56; *P* < .001, with 2133 patients).

In our study analysis, there was no difference in survival to middle-term follow-up in the setting of NSTEMI OHCA between early CAG and delayed or no CAG. This was not consistent with previous studies that did show a survival benefit with early CAG in this patient population. The study by Khan et al^[27]^ reported that the use of early CAG was associated with decreased short-term mortality (OR = 0.46; 95%CI = 0.36–0.56; *P* < .001 in eight studies with 2133 patients) and long-term mortality (OR = 0.59; 95%CI = 0.44–0.74; *P* < .001). In our study, the survival to middle-term follow-up was defined as 30 to 90 days after hospital discharge in patients with NSTEMI following OHCA. This time difference may be the explanation as to why our study results were inconsistent with the results from Khan et al.^[27]^ In addition, our study also suggests that long-term follow-up survival in the setting of NSTEMI OHCA was not investigated and results were not reported in two new studies.^[1,17]^

Recently, Patterson et al^[14]^ published the pilot results of a randomized controlled trial investigating the outcomes of an early invasive approach in NSTEMI following OHCA. These findings support the feasibility and acceptability of conducting a large-scale randomized controlled trial of expedited transfer to CAG following OHCA to address a remaining uncertainty in post-arrest care (30-day mortality [Intervention 9/18, 50% vs Control 6/15, 40%; *P* = .73], cerebral performance categories 1 and 2 [Intervention 9/18, 50% vs Control 7/14, 50%; *P* > 0.99]). Lemkes et al^[1]^ published that among patients who had been successfully resuscitated after OHCA with NSTEMI, immediate CAG was not found to be better than delayed CAG with respect to overall survival at 90 days (OR, 0.89; 95%CI, 0.62–1.27; *P* = .51). These findings currently constitute the only two randomized data. There are some differences in the results of the Lemkes trial and those of previous studies: selection bias and patient population. The vast majority of patients in the Lemkes trial had stable coronary artery lesions, and thrombotic occlusions were encountered in only 5% of patients. Therefore, future randomized controlled trials should attempt to deliver standardized post-resuscitation care that varies only in provision of early CAG. Several other large-scale randomized controlled trials investigating outcomes of early CAG in NSTEMI after OHCA are currently underway.^[28,29]^ Randomization of OHCA NSTEMI patients to an early invasive/intervention arm will have significant implications for the concomitant delivery of other goal-directed therapies.

The time window of CAG in patients with NSTEMI following OHCA was unclear. According to current practice recommendations, CAG in alert patients with STEMI should occur emergently.^[4]^ Some studies have confirmed the benefits seen in very early (<2 h) and intermediate-early (<6 h) CAG, and similar studies were reported in the literature that included “early” CAG up to 24 h. The definitions and performance of “early” and “late” CAG varied across the pooled studies and could have contributed to the variability in our results. A coordinated response to care has been suggested to address these issues simultaneously^[30,31]^ and might allow for standardization of CAG timing while helping to define early CAG metrics in patients with NSTEMI.

## 5 Limitations

The risk of observational bias always exists, although we did search a range of international and Chinese databases without language constraints, and Egger's and Begg's tests suggested no significant risk of observational bias. Although large, our total sample of 1599 individuals with early CAG and 2287 controls may still be subject to random error. Because our meta-analysis examined ethnically diverse populations from various countries, heterogeneity may have affected our results. Future work regarding an early CAG protocol should apply this new criterion.

## 6 Conclusions

Despite these limitations, our present meta-analysis provides important documentation that there are survival benefits conferred by early CAG in the setting of NSTEMI OHCA. These findings should be extended to large, multi-site randomized controlled trials and observational studies. The results may inspire clinicians to focus on early CAG in the setting of NSTEMI OHCA.

## Author contributions

Meng-Chang Yang, Wu Meng-Jun, Xu Xiao-Yan and Kevin L. Peng wrote the paper. Yong G, Peng and Ru-Rong Wang designed the study, reviewed, and edited the manuscript.

## Correction

When originally published, Meng-Chang Yang's name appeared incorrectly as Yang Meng Chang and Ru-Rong Wang's name appeared incorrectly as Wang Ru-rong.

## Abbreviations

Abbreviations: CAG = coronary angiography, OHCA = out-of-hospital cardiac arrest, PCI = percutaneous coronary intervention, STEMI = ST segment elevation myocardial infarction.
